# A longitudinal examination of US teen childbearing and smoking risk

**DOI:** 10.4054/DemRes.2018.38.24

**Published:** 2018-02-21

**Authors:** Stefanie Mollborn, Juhee Woo, Richard G. Rogers

**Affiliations:** 1 Department of Sociology and Health and Society Program, Institute of Behavioral Science (IBS), University of Colorado Boulder, USA; 2 Department of Sociology and Problem Behavior and Positive Youth Development Program, Institute of Behavioral Science (IBS), University of Colorado Boulder, USA; 3 Department of Sociology and Population Program, Institute of Behavioral Science (IBS), University of Colorado Boulder, USA

## Abstract

**BACKGROUND:**

Teenage motherhood and smoking have important health implications for youth in the United States and globally, but the link between teen childbearing and subsequent smoking is inadequately understood. The selection of disadvantaged young women into early childbearing and smoking may explain higher smoking levels among teen mothers, but teen motherhood may also shape subsequent smoking through compromised maternal depression or socioeconomic status, and race/ethnicity may condition these processes.

**OBJECTIVE:**

This study examines the relationship between US teen childbearing and subsequent daily smoking, accounting for prior smoking and selection processes related to social disadvantage. Analyses investigate whether socioeconomic status and depression in young adulthood explained any relationship between teen childbearing and daily smoking, as well as examining racial/ethnic heterogeneity in these processes.

**METHODS:**

Multivariate binary logistic regression analyses employ the National Longitudinal Study of Adolescent to Adult Health (Add Health; N = 7,529).

**RESULTS:**

The highest daily smoking prevalence occurred among non-Hispanic White teen mothers, with lower prevalence among Hispanic and non-Hispanic Black teen mothers. Compared to other women, teenage mothers are 2.5 times as likely to smoke daily in young adulthood. Their greater likelihood of daily smoking is due in part to selection and is also mediated by socioeconomic status in ways that differ by race/ethnicity.

**CONCLUSIONS:**

The findings suggest that preventing teen pregnancy or ameliorating its socioeconomic consequences may decrease daily smoking in this vulnerable population. Reducing teen smoking, especially during pregnancy, could improve teen, maternal, and infant health and thereby increase US health and longevity.

**CONTRIBUTION:**

This study provides new, nationally representative information about selection, mediation, and heterogeneity processes in the relationship between teen childbearing and subsequent smoking.

## 1. Introduction

Both cigarette smoking and teen childbearing are considered urgent contemporary health issues in US society and other countries around the world. Despite recent declines, compared to peer countries (Sedgh et al. 2015), the United States has a relatively high teen birth rate at 22.3 births per 1,000 girls aged 15–19 in 2015 (Martin et al. 2017). In high-income nations, smoking is the largest cause of premature mortality (Centers for Disease Control and Prevention 2008; Rogers et al. 2005), and “smoking during pregnancy is considered the largest preventable cause of fetal and infant ill health and death” (Delpisheh, Brabin, and Brabin 2006: 389). Smoking typically begins in late adolescence: Cigarette use in the past month increased from just 4.0% among 15-year-olds to 23.8% among 19-year-olds in United States in 2015 (Center for Behavioral Health Statistics and Quality 2016). Given the public health concerns around teen pregnancy and cigarette smoking and calls for research on their association (e.g., McDermott and Graham 2006), it is surprising that relatively little research has been conducted. Our study addresses that gap by estimating the relationship between teen childbearing and subsequent daily smoking.

As with other potential consequences of teen parenthood, smoking’s relationship to teen childbearing may be substantially complicated by selection processes and heterogeneity of effects. We seek to fill gaps in the literature by unpacking these processes. Preexisting social disadvantages based on characteristics such as socio-economic status, race/ethnicity, and geographic location shape young women’s selection into both teen childbearing (Erdmans and Black 2015; Furstenberg 2003; Kearney and Levine 2015; Wodtke 2013) and smoking (Chassin et al. 1996; Lawrence, Pampel, and Mollborn 2014). In other words, young women who become smokers and those who become teen mothers are not evenly distributed throughout the population, and in many cases they disproportionately come from the same segments of the population. Because teen motherhood and smoking are strongly related to social disadvantage, they are likely associated with each other in ways that may not be causal. Furthermore, there may be heterogeneity in the relationship between teen childbearing and subsequent smoking, with different associations for different groups. This study focuses on race/ethnicity as a source of heterogeneity because it is strongly associated with both teen motherhood and smoking in complex ways (Dennis and Mollborn 2013; Lawrence, Pampel, and Mollborn 2014; Williams et al. 2015).

Using nationally representative survey data from the National Longitudinal Study of Adolescent to Adult Health (Add Health) that followed US teens until their late twenties or early thirties, this study estimates the relationship between teen childbearing and daily cigarette smoking at multiple points in the early life course. Because they represent a smaller proportion of the population than teen mothers and are less likely to live with their children (Mollborn and Lovegrove 2011), teen fathers are a distinct and hard-to-reach population that may have different smoking implications and that is not studied here. Our longitudinal data allows us to generalize findings to a recent US cohort and to establish time order between teen childbearing and daily smoking. We assess the roles of specific selection processes, explore potential mediators of the relationship between teen motherhood and smoking, and investigate racial/ethnic heterogeneity in these relationships and processes. Each of these goals is a new contribution to the literature. Understanding not only how teen motherhood and smoking are related to each other, but the extent to which those relationships may be driven by selection and how heterogeneous they are across different groups is important for social policy. If the two phenomena are potentially causally related at least in some groups, then policy efforts to reduce one might also ameliorate the other.

**Figure 1:**
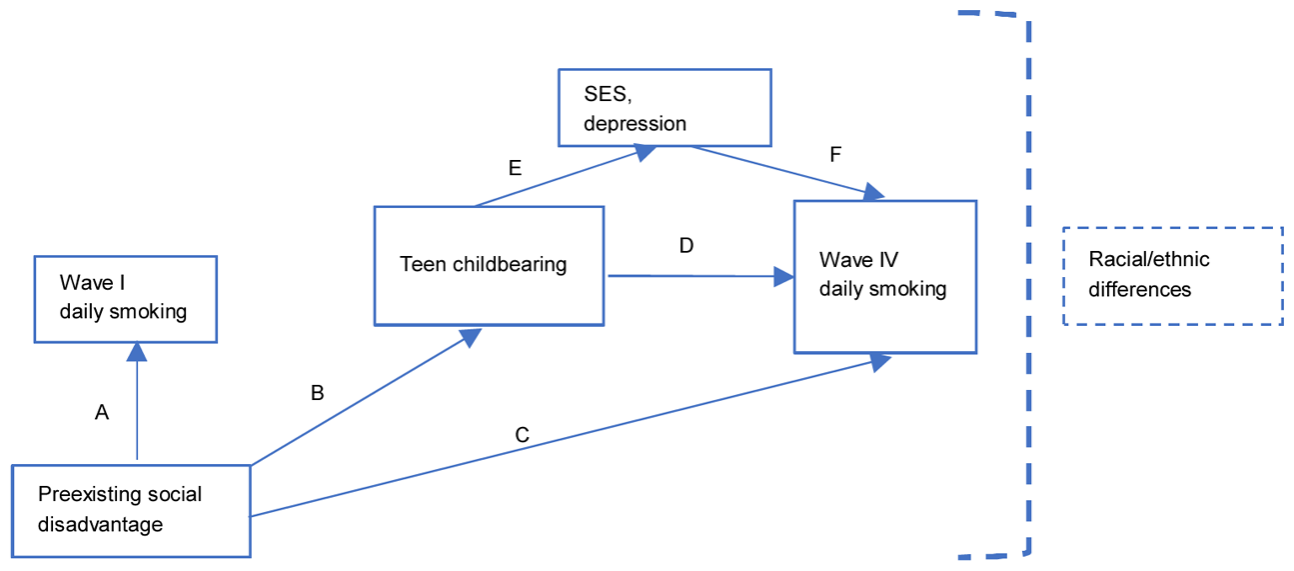
Conceptual model Does teen childbearing predict daily smoking in young adulthood in a nationally representative longitudinal study of women (Figure 1, path D)?If teen childbearing is associated with subsequent smoking, is this relationship explained by the selection of socially disadvantaged teens into smoking and teen childbearing (paths A–C)?After accounting for selection processes, do socioeconomic status and depression in young adulthood mediate any remaining relationship between teen childbearing and subsequent daily smoking (paths D–F)?Are there racial/ethnic differences in the association between teen childbearing and subsequent smoking or in its selection and mediation processes?

Our goal is to disentangle the potentially complicated interplay between teen childbearing and smoking by examining their relationship at different time points, the role of selection, potential mediators, and racial/ethnic heterogeneity in each of these phenomena. We explore four primary research questions, based on our conceptual model shown in Figure 1.

## 2. Background

### 2.1 Teen motherhood and cigarette smoking

US teen childbearing rates peaked in the 1950s, but teen motherhood emerged as a perceived social problem in later decades as most teen births became nonmarital (Furstenberg 2003). A large, longstanding literature has examined the consequences of teen childbearing for a variety of short- and long-term life outcomes (e.g., Taylor 2009). Some influential studies have found that the documented negative consequences of teen childbearing are mostly or entirely due to the selection of disadvantaged girls into early motherhood (Geronimus and Korenman 1993; Hoffman 1998; Turley 2003). But most researchers now attribute modest negative effects to teen motherhood (Fletcher and Wolfe 2009; Kane et al. 2013), with heterogeneity in these effects for different groups (Diaz and Fiel 2016; Williams et al. 2015).

Here, we consider the medium-term outcome of daily smoking in young adulthood (Figure 1, path D). Research on the relationship between teen childbearing and later smoking is sparse. Teen mothers-to-be have higher rates of prenatal smoking than most older age groups (Lu, Tong, and Oldenburg 2001; Mathews 2001; Ventura et al. 2003), even though they are disproportionately likely to quit smoking while pregnant (Colman and Joyce 2003; Curtin and Mathews 2016). White US teen mothers had a particularly high prenatal smoking prevalence of 34% in 2000–2001, compared to 9% for African American and 11% for Hispanic teen mothers (Dennis and Mollborn 2013). White teen mothers’ high levels of smoking partially explained their children’s elevated risk for low birthweight compared to children of older White mothers (Dennis and Mollborn 2013). After childbearing, evidence from the United Kingdom and Australia suggests that teen mothers have an increased likelihood of smoking (Graham et al. 2006; Webbink, Martin, and Visscher 2008). Young mothers in a high-risk New York sample were more likely to be current smokers than those who were childless (Stueve and O’Donnell 2007). Teen mothers are more likely than older women to resume smoking after childbirth (Colman and Joyce 2003), making the otherwise protective effect of childbearing weaker for this group.

### 2.2 Selection into smoking and teen childbearing

Any relationship between teen childbearing and smoking may be caused by preexisting social disadvantages underlying both behaviors (Figure 1, paths A–C). By comparing pregnant teens who miscarried with those who gave birth, as well as estimating family fixed effects, Fletcher (2011) found that such selection processes accounted for the initial relationships between teen motherhood and smoking in the short and long term. Three selection factors that may be particularly important for explaining observed associations between teen childbearing and smoking are preexisting social class, depression, and preexisting smoking. Cumulative social class across childhood predicts prenatal smoking among women (Spencer 2006), as well as teen childbearing (Quinlivan et al. 2004). Depression is related to smoking before pregnancy (Graham et al. 2006; Hanna, Faden, and Dufour 1994), and among severely depressed girls living in poverty it also predicts teen childbearing (Mollborn and Morningstar 2009). Teen pregnancy is part of a constellation of related adolescent risk behaviors that may have implications for its relationship with smoking. Because they have often experienced early sexual intercourse, many teenage parents are expected to evidence a “syndrome of problem behavior” predicted by social disadvantage, being more likely than other adolescents to engage in unhealthy behaviors such as problematic alcohol use, marijuana use, and delinquency (Donovan, Jessor, and Costa 1988). Thus, because of social disadvantage underlying both behaviors, girls who have teen births may have already been more likely to smoke. In a high-risk local urban sample, women who had become mothers by about age 20 were more likely to have smoked in the past (Stueve and O’Donnell 2007). Evidence from the United Kingdom suggests that teen childbearing can compound smoking risk stemming from earlier childhood disadvantages (Graham, Hawkins, and Law 2010), which implies a blend of selection and causality.

### 2.3 Explanations for a relationship between teen childbearing and smoking

If selection processes do not fully account for the relationship between teen childbearing and subsequent smoking, what potential mediators could explain this association (Figure 1, paths D–F)? Teen motherhood is a stigmatized and stress-inducing experience for many reasons, including the violation of age norms, potential disruption to human capital formation, its association with depression, and the stress of motherhood more generally (Falci, Mortimer, and Noel 2010; Kane et al. 2013; Larson 2004). Cigarette smoking is a known strategy for coping with stress that tends to be classed and raced (Chassin et al. 1996; Lawrence et al. 2014). In a small qualitative study of teen mothers, Lawson (1994) found that a majority had already begun smoking between 10 and 11 years old. They reported reasons why they continued smoking during and after pregnancy, perceiving that the short-term benefits of smoking, such as relief from anxiety and weight control after childbearing, outweighed longer-term health risks.

The survey we analyze cannot assess these potential mediators, but we do evaluate others. We focus on two prevalent phenomena among teen mothers (see above) that may increase subsequent smoking, possibly mediating any relationship between teen childbearing and subsequent smoking: low socioeconomic status attainment and elevated depression symptoms. We argue that teen childbearing may elevate depression and reduce socioeconomic attainment (path E), which could in turn increase the likelihood of daily smoking (path F), explaining why teen childbearing predicts daily smoking (path D). The relationships between teen childbearing and both depression and reduced socioeconomic attainment have been well studied and are described above. Prenatal smoking is more prevalent among women who have lower levels of education and income and those who have mood disorders (Erlingsdottir et al. 2014; Gilman et al. 2008; Hanna, Faden, and Dufour 1994; Lu, Tong, and Oldenburg 2001; Maxson et al. 2012; Zimmer and Zimmer 1998). In adulthood, less educated people are less likely to quit smoking (Chassin et al. 1996; Pickett et al. 2003).

### 2.4 Racial/ethnic heterogeneity in relationships between teen childbearing and smoking

Women’s cigarette smoking is patterned by race/ethnicity. White women begin smoking at younger ages than Black women (Geronimus, Neidert, and Bound 1993; Moon-Howard 2003) but are more likely to quit and do so at younger ages (Geronimus, Neidert, and Bound 1993). Using Add Health data, Chen and Jacobson (2012) and Lawrence and colleagues (2014) found that among African Americans, smoking rates started low but increased with age, resulting in the highest smoking rates of any racial group by their early thirties. There was an inverse U-shaped relationship between smoking and age for White, Asian American, and Hispanic respondents, with smoking rates increasing throughout adolescence and peaking in their mid-twenties. The age-related increase and decrease around this peak were particularly sharp for Whites (Chen and Jacobson 2012), and analyses of other data have found that this cessation among Whites is the main explanation for racial convergence in smoking prevalence over the life course (Pampel 2008).

Fertility complicates racialized smoking uptake and cessation patterns. A greater proportion of White women smoke during pregnancy compared to Black women (Arnold et al. 2001; Mathews 2001; Ventura et al. 2003). Adjusting for selection using within-family comparisons, Geronimus and Korenman (1993) found suggestive evidence that White teen mothers were more likely than their sisters to smoke during pregnancy, but the same was not true for African American teen mothers. Although childbearing generally lowers the likelihood of subsequent smoking (Pampel, Mollborn, and Lawrence 2014; Staff et al. 2010), this is likely not true for African American teen mothers, as their smoking rates continue to increase throughout their twenties and thirties after they have given birth. Unlike other groups, African American women who start smoking in adulthood are not more likely to stop smoking than those who started in their teens (Thompson, Moon-Howard, and Messeri 2011).

Teen childbearing is heavily patterned by race/ethnicity. Teen birth rates are considerably higher among Hispanics and African Americans than among Whites, at 34.9, 31.8, and 16.0 births per 1,000 women aged 15–19 respectively (Martin et al. 2017). Thus, because they represent a smaller and more disadvantaged proportion of the White population, White teen mothers are a more selected, atypical subpopulation than are Hispanic or Black teen mothers. Information on the role of race/ethnicity for the consequences of teen childbearing is limited. Mollborn (2010) found that race/ethnicity did not predict high school completion among US teen mothers and fathers. In contrast, Williams and associates (2015) identified negative implications of teen birth for self-rated health at midlife compared to giving birth after age 25 for Black but not for White or Hispanic women. Because it structures both teen childbearing and smoking, race/ethnicity may be a source of heterogeneity in their relationship.

### 2.5 Analytic plan

We capitalize on the longitudinal, nationally representative Add Health data to observe daily smoking before pregnancy and into young adulthood, several years after a teen birth. The core relationship we estimate is between teen childbearing and the odds of daily smoking in young adulthood. To answer the research questions, we estimate the influence of selection processes on this relationship, test for mediation of the adjusted relationship by socioeconomic attainment and depression symptoms, and examine whether all these relationships differ by the teen mother’s race/ethnicity. Addressing these goals requires longitudinal survey data following a large sample of young women over time, from before to well after the experience of teen childbearing, and comparing them to peers who did not experience a teen birth.

## 3. Methods

### 3.1 Data

We investigate the relationship between cigarette smoking in young adulthood and experiences of teenage motherhood using data from Add Health (Harris et al. 2009), a nationally representative survey of US adolescents. Add Health collected data on a variety of health-related subjects throughout four waves (wave 1 from September 1994 through December 1995; wave 2 from April 1996 through August 1996; wave 3 from August 2001 through April 2002; wave 4 from January 2008 through February 2009).

In wave 1, 132 schools (80 high schools and 52 middle schools) were sampled to represent US schools in regard to region of county, urbanicity, size, type, and ethnicity. More than 70% of these schools participated in the study, and schools that refused to participate were replaced by schools within the same community. A subsample of students in each school and their primary parent (typically the mother) completed an interview. Students were followed up for additional interviews in the next three waves (graduating seniors and some others were not re-interviewed at wave 2). Some populations were oversampled, yet sampling weights, region, and school identifier allow researchers to represent the national population of adolescents.

For this study, we mainly used data from waves 1 and 4. Wave 4 data is the most recent information on the study participants, who were settling into young adulthood (ages 24 to 32) at the time of the interview. We used it to measure our outcome variable (daily smoking in young adulthood), as well as respondents’ characteristics in young adulthood that may mediate the relationship between teen motherhood and the likelihood of smoking in young adulthood. Wave 1 data provide information on the respondents’ teenage years (grades 7 through 12), including their family backgrounds. We used this data for our selection variables, which may generate a spurious relationship between teenage motherhood and smoking in young adulthood. We used wave 2 and 3 data only to measure daily smoking, which was needed for descriptive analyses of smoking across the four waves but was not used in multivariate models.

Our sample was limited to women, identified using respondents’ reports of biological sex in waves 1 and 4. Among them, 7,874 respondents who participated in Waves 1 and 4 and who were not missing sampling weights, region, or school identifier were eligible for analysis. We omitted those who gave birth before or were pregnant at wave 1 to establish time order between smoking at wave 1 and teen childbearing, leaving a final sample of 7,529.^4^ Following the recommendation of Allison (2002), we used all analysis variables to impute missing values on independent and dependent variables, which retains all cases, reduces bias resulting from listwise deletion of missing cases, and produces unbiased estimates (Schafer 1999; Sterne et al. 2009). Missing values were imputed by the ‘mi impute chained’ command, and all analyses were conducted using Stata statistical software (StataCorp 2011).^5^

### 3.2 Variables

#### 3.2.1 Daily smoking at wave 4

Given the high prevalence of social smokers among adolescents and young adults, who smoke occasionally in certain social settings (Schane, Glantz, and Ling 2009; Song and Ling 2011), we focused our analyses on daily smokers, who may be at greater health risks than social smokers. Studies categorize current smokers into daily and intermittent smokers and define intermittent smokers as those who smoke “some days” (Lindström 2003; Trinidad et al. 2009). Although intermittent smoking can be a chronic pattern of cigarette use, it is also perceived as a transitional phase (from nonsmoking to daily smoking and vice versa; Berg et al. 2013). Adolescent and young adult intermittent smokers tend to be experimental or social smokers and may not transition to daily smokers (Wetter et al. 2004). Daily smokers, on the other hand, may have established their smoking habits and are less likely to quit, thus drawing greater attention for health interventions.

We defined daily smokers using respondents’ self-reports of smoking during the past 30 days. At wave 4, an initial question asked respondents whether they had ever smoked an entire cigarette. Those answering ‘yes’ were then asked how many days during the past 30 days they had smoked. Our study classified those answering 30 days as daily smokers (coded 1), while non-daily smokers were those who either had never smoked an entire cigarette or smoked during fewer than 30 days in the past month. For similar coding, see Johnson and Hoffman (2000) and Kandel et al. (2004).

#### 3.2.2 Teen childbearing

We defined a teen mother as a woman who had her first child before the month of her twentieth birthday. Using wave 4 retrospective fertility histories (the recommended source of fertility information in Add Health), we subtracted each respondent’s birth month and year from the pregnancy end date to measure the mother’s age at her first birth. We dropped two women who reported giving birth before age 11, so the sample’s teen mothers were ages 11–19 at first birth. Among the sample (N = 7,529), 4,014 women had a live birth, with 1,002 teen mothers and 3,012 adult mothers who gave birth after age 20. The other 3,515 women had not given birth by wave 4.

#### 3.2.3 Sociodemographic variables

We chose all independent variables (demographic characteristics, selection factors, wave 1 smoking, and mediators) because of their possible relationships to smoking or teenage motherhood. Multicollinearity among the variables was not a concern in the models. As a control, respondents’ age at wave 1 was calculated by subtracting their birth month and year from the wave 1 interview date. We measured respondents’ race/ethnicity, a potential source of heterogeneity, by four categories: Hispanic, non-Hispanic White, Black, and other race. We combined American Indian/Alaska Native and Asian/Pacific Islander as “other race” and excluded them from the analysis of racial/ethnic comparisons due to their small sample size of teen mothers. Respondents’ nativity was coded from a question asking if they had been born in the United States (US-born = 1; foreign-born = 0).

We included several selection variables, all measured at wave 1. First, student-reported grade point average (GPA) for four subjects was averaged into a four-point scale (D or lower = 1, C = 2, B = 3, A = 4) and recoded into a series of indicator variables (less than 3, 3 to 3.49, and 3.5 or more). Family socioeconomic status was measured by parents’ education and parent-reported household income. We recoded parent reports of earned degrees into approximate years of education.^6^ The highest education levels of the mother and her spouse/partner were averaged. If the spouse’s education was missing from the parent report, we substituted the adolescent respondent’s report of his education level. Lacking that, the mother’s education was used for both. If no parent completed the survey, the adolescent respondent’s report of both parents’ education levels was substituted. Cubbin and associates (2005) found 75% agreement between parents’ and adolescents’ reports of parental education levels when data for both were available. Following Cubbin et al. (2005), we adjusted for the number of people in the household to create an indicator variable that represents parent-reported household income as a percentage of 1994 federal poverty thresholds (0 to 100, 101 to 200, 201 to 300, 301 to 400, and greater than 400%). Respondents’ family structure was coded into a series of indicator variables after Harris (1999): living with both biological parents, other types of two-parent families, a single mother, a single father, and other family structures. Religious service attendance was measured by frequency and coded into a series of indicator variables (never/no religion, less than once a month, once a month or more but less than once a week, and at least once a week).

We also measured respondents’ wave 1 levels of depression symptoms and delinquency. The measure of depression symptoms used a subset of questions from the Center for Epidemiologic Studies Depression Scale (CES-D; Radloff 1977). Respondents were asked 19 questions about how often they had particular feelings in the past week (0 = never or rarely, 1 = sometimes, 2 = a lot of the time, 3 = most or all of the time), and the average of the 19 questions was used as a depression symptoms scale at wave 1 (alpha scale reliability coefficient = 0.91). In addition, respondents were asked 15 questions about how often they engaged in delinquent acts (0 = never, 1 = one or two times, 2 = three or four times, 3 = five or more times). The delinquency scale represents the respondents’ averaged answers to the 19 questions after reverse coding positive items (alpha scale reliability coefficient = 0.84). Because the depression and delinquency scales were highly skewed, we logged both scales after adding 1. Lastly, we included a dichotomous indicator of respondents’ reports of having ever had vaginal intercourse by wave 1. Because women who were pregnant were excluded from the sample, this was preexisting sexual activity that could not have led to the teen birth.

Respondents’ daily smoking status at wave 1 was another selection measure. Since we excluded respondents who gave birth before or were pregnant at wave 1, daily smokers at wave 1 would have started smoking before they became pregnant. In wave 1, the initial question asked respondents if they had ever tried cigarette smoking, even just one or two puffs. Those who had tried smoking and smoked 30 days in the past month were categorized as daily smokers (1), whereas those who had never tried smoking, never smoked an entire cigarette, or smoked less than 30 days in the past month were coded as 0.

For descriptive analysis of smoking of each racial/ethnic group over time, daily smoking in waves 2 and 3 was also measured. Wave 2 smoking measures were different from the other three waves, as respondents were asked about their smoking activity since the month of the wave 1 interview. We defined daily smokers as those who reported having tried smoking since the last interview (wave 1) and smoking 30 days in the last month. At wave 3, we defined daily smokers as those who reported having smoked an entire cigarette and smoking 30 days during the past month.

#### 3.2.4 Potential mediators

Respondents’ socioeconomic status attainment (education attainment, income, and home ownership) and levels of depression symptoms, measured at wave 4, were considered potential mediators of the relationship between smoking and teenage motherhood. Teen childbearing has been shown to compromise mental health and educational and other socioeconomic attainment (see above), which may in turn increase smoking. Each respondent’s highest level of education achieved was recoded into approximate years from a categorical measure (earned degrees, recoded as in wave 1). Wave 4 household income is coded as a percent of the 1997 federal poverty thresholds, adjusting for household size (recoded as in wave 1). We measured home ownership by a dichotomous indicator of whether the respondent’s house, apartment, or residence is owned by themselves or their partner versus not. For the modified CES-D depression symptoms scale at wave 4, respondents were asked 10 questions about how often they had particular feelings in the past week (0 = never or rarely, 1 = sometimes, 2 = a lot of the times, 3 = most of the time or all of the time), and we averaged their responses after reverse coding positive items (alpha scale reliability coefficient = 0.84).

### 3.3 Analysis

All analyses accounted for clustering and stratification in the survey design and used Add Health’s longitudinal probability weights to produce nationally representative findings assessing the model laid out in Figure 1. First, we calculated means for all variables used in analysis, comparing wave 4 daily smokers and others. Next, we estimated binary logistic regression models to examine the relationship between teenage motherhood and the likelihood of daily smoking at wave 4, controlling for other variables and comparing teen mothers to all other women. Supplemental models, which separated the comparison group into nonmothers at wave 4 (the group least likely to smoke) and mothers whose first birth occurred after their teenage years, did not produce substantively different conclusions, except for some specific changes to significance levels in models disaggregated by race/ethnicity. The baseline model compared the likelihood of daily smoking for teen mothers and all other women, controlling for respondents’ demographic characteristics, such as age, race/ethnicity, and nativity. Next, we added selection variables (model 2) and daily smoking at wave 1 (model 3) to tease out selection processes surrounding teen childbearing and smoking. Model 4 added potential mediators of the relationship between teenage motherhood and the likelihood of daily smoking in early adulthood (mediation tests were conducted using the logic laid out by Baron and Kenny 1986). Subsequent models included the 7,036 women in the sample who were Hispanic or non-Hispanic Black or White. We introduced interactions into the full model (model 4) to investigate whether the relationship between teenage motherhood and smoking differed across racial/ethnic groups. We also examined the smoking trajectories of teen mothers versus all other women, aggregated by racial/ethnic groups (non-Hispanic White, non-Hispanic Black, and Hispanic), to better understand the interaction effects. All analyses used Stata’s complex survey design commands (StataCorp 2011). We present odds ratios, or exponentiated coefficients, for the logistic regression models.

## 4. Results

### 4.1 Associations between teen childbearing and smoking

Table 1 reveals that teen childbearing and subsequent wave 4 daily smoking are strongly related (path D in Figure 1). Teen mothers made up 21% of daily smokers at wave 4, compared to 11% of nonsmokers and 14% of the overall sample. Table 2, model 1 estimated this relationship after controlling for age, race/ethnicity, and nativity. Becoming a teen mother before wave 4 was associated with 1.5 times higher odds of smoking at wave 4 compared to other women (odds ratio = 2.53).

### 4.2 Selection processes

Table 1 shows that sociodemographic factors (such as socioeconomic status, family structure, and academic achievement) shaped the selection of women into wave 4 smoking status (Figure 1, path C). Supplemental descriptive analyses found similar selection processes for wave 1 daily smoking and teen childbearing as well (paths A and B). Table 2, model 2 adjusts for various selection factors predating teen mothers’ pregnancies, including academic achievement, family socioeconomic status, family structure, religious attendance, depression symptoms, delinquency, and past sexual activity. Including these covariates reduced the association between teen childbearing and later smoking (path D) by nearly two-thirds to 0.6 times higher odds of smoking among teen mothers (odds ratio = 1.57), but it was still significant. Another important selection factor potentially underlying teen childbearing and wave 4 daily smoking was wave 1 daily smoking. Adjusting for this measure in model 3 further attenuated teen mothers’ likelihood of smoking to 1.47 times as high as other women’s. Thus, the selection processes measured in this study accounted for most but not all of the relationship between teen childbearing and subsequent smoking.

### 4.3 Mediation of the teen childbearing-smoking relationship

Table 2, model 4 shows that introducing socioeconomic attainment and depression into the model (Figure 1, path F) resulted in the coefficient for teen motherhood (path D) losing statistical significance. In testing for mediation using the criteria outlined by Baron and Kenny (1986), we found that educational attainment and income-to-needs ratio together mediated the teen childbearing-daily smoking relationship in path D. Supplemental models showed that teen childbearing did not significantly predict home ownership or depression symptoms (path E), so they did not qualify as mediators. In a supplemental model in which only education and income were included as mediators, the teen childbearing odds ratio was 1.14 and was not statistically significant. Thus, education and income were the mediators that explained why teen childbearing was positively related to subsequent smoking: Teen childbearing predicted reduced subsequent household income and educational attainment, which were associated with an increased likelihood of daily smoking.

### 4.4 Racial/ethnic heterogeneity in processes

**Figure 2:**
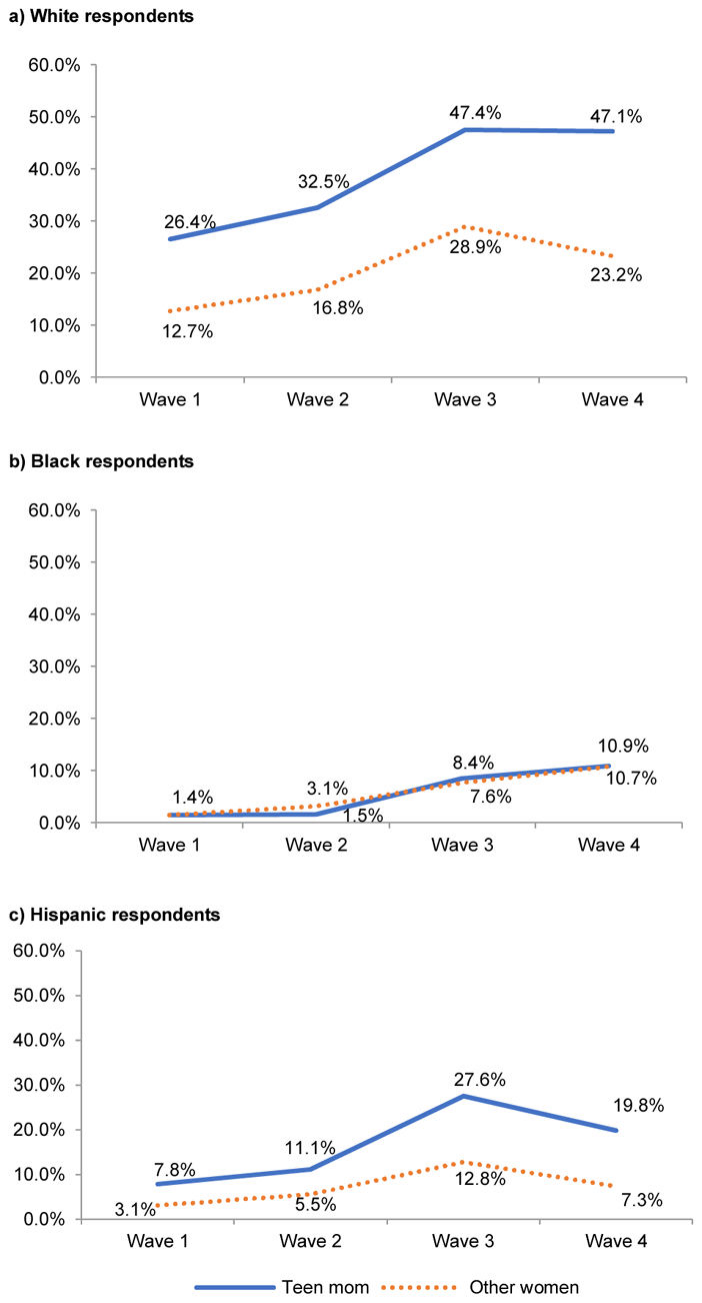
Smoking prevalence among respondents by teen mother status, disaggregated by race/ethnicity *Source:* National Longitudinal Study of Adolescent Health (1995). *Note:* Analyses account for sample design effects (weighting, stratification, and clustering).

The proportion of teen mothers varied across racial/ethnic groups: 11.3% of White (n = 4163), 19.9% of Black (n = 1681), and 19.1% of Hispanic (n = 1191) women were teen mothers. Figure 2 shows considerable racial/ethnic variation in the unadjusted relationship between teen childbearing and daily smoking across adolescence and young adulthood. The figure reports smoking prevalence at each wave of the survey for teen mothers and other women. Wave 1 prevalence predated teen pregnancies in the sample, so it illustrates the selection of daily smokers into teen childbearing. This selection was substantial among White women, with 26.4% of future teen mothers smoking compared to 12.7% of other women. This was true to a lesser extent for Hispanics at 7.8% and 3.1% respectively, but there were no differences between Black future teen mothers and other Black women. Disparities in smoking between teen mothers and other women widened with age for White women, particularly between wave 3 (after nearly all women had become teen mothers) and wave 4. Between these waves, White teen mothers’ smoking prevalence held steady, while it decreased for other White women. This widening also occurred for Hispanics, but it happened primarily between waves 2 and 3 (the period during which most of the teen births occurred for all racial/ethnic groups). There was little difference in smoking between Black teen mothers and other Black women at any wave.

At all waves, there were large racial/ethnic differences in smoking prevalence, with the highest among Whites, the lowest among African Americans, and Hispanics in between. By wave 4, 47.1% of White teen mothers were daily smokers, compared to 19.8% for Hispanic teen mothers and 10.9% for Black teen mothers. Thus, even White women who never became teen mothers still had a smoking prevalence (23.2%) that was higher than any group of Black or Hispanic women.

Given the racial/ethnic differences in the gaps in smoking prevalence between teen mothers and others portrayed in Figure 1, it is perhaps unsurprising that Table 2, model 5 identified significant differences by race/ethnicity in the relationship between teen motherhood status and the likelihood of later smoking (Figure 1, path D). To explore racial/ethnic differences in selection and mediation processes, Table 3 presents the same first four models as Table 2 but disaggregated by race/ethnicity for White, Black, and Hispanic women. There was no significant relationship between teen childbearing and smoking among Black women in any model. Only for White women was there a significant relationship between teen motherhood and wave 4 smoking at the p<.05 level after controlling for social background in model 2. For Hispanics, this relationship was larger in magnitude than for White women (an odds ratio of 1.94 compared to 1.68) but was only significant at the p<.10 level. This marginally significant relationship was explained by the socioeconomic mediators, as it was for White women. Supplementary models showed that for both Hispanic and White women as in Table 2 for all women, education and income mediated the relationship.

## 5. Discussion

We found a strong association between teenage childbearing and subsequent daily smoking: Teen mothers were 1.5 times as likely as other women to smoke daily at wave 4, controlling for demographic factors and previous smoking. This greater likelihood of daily smoking was due in large part to selection factors. Controlling for academic achievement, family structure and socioeconomic status, religiosity, delinquency, previous sexual activity, and preexisting smoking substantially reduced the association between teen motherhood and subsequent smoking. Achieved socioeconomic status mediated the remaining relationship: Teen childbearing reduced women’s educational attainment and household income, which in turn increased their likelihood of smoking.

Teen mothers are disadvantaged in part because of lower socioeconomic status and riskier behaviors, including smoking. And their smoking during or after pregnancy may result in further disadvantage. Smoking during pregnancy may lead to lower birthweight babies who require more personal and medical care, which may further exacerbate the disadvantaged positions of their mothers. Furthermore, parental smoking can expose infants and children to secondhand smoke, which can increase their risk of disease and death (US Department of Health and Human Services 2006). Thus, we have identified one pathway through which socioeconomic and health disadvantage may accumulate among young mothers and contribute to the intergenerational transmission of poor health: Social disadvantage is associated with becoming a teen mother, which predicts lower socioeconomic attainment, which is related to increased risk of smoking, which can increase the risk of preterm and low weight births and of infant and child disease and mortality. These risky infants and children may require expensive and time consuming medical care and may experience developmental delays that further stress the mother and reduce her educational progress, perpetuating the vicious cycle of intergenerational inequality.

There were substantial racial/ethnic differences in the relationship between teen childbearing and smoking, as well as in the selection and mediation processes underlying the relationship. Smoking prevalence was highest among non-Hispanic Whites and lower among non-Hispanic Blacks and Hispanics. It is noteworthy that the racially advantaged group had the highest smoking prevalence rate. White teen mothers’ high smoking prevalence rates may be related to teen mothers in this racial/ethnic group being a more highly selected population and also represents a unique risk for this subpopulation. Teenage childbearing did not predict subsequent smoking among Black women, was marginally significant and mediated by income and education among Hispanics, and was large, significant, and mediated by education and income among Whites. Although it is important to reduce smoking among all young mothers, it may be especially important to reduce smoking among young White mothers, even years after childbirth. Gittelsohn and colleagues (2001) studied adolescents in Baltimore, finding that compared to African Americans, Whites perceived more parental permissiveness toward smoking and were more likely to smoke to fit in with their peers. Thus, antismoking programs could target teens’ social networks, including family members and friends (Christakis and Fowler 2008).

Effective social and health policies can break the vicious cycle of intergenerational inequality. Policy makers can find ways to reduce risky behaviors such as smoking and unprotected sex during youth and adolescence; provide school-, community-, and clinic-based smoking cessation programs; reduce teenage childbearing; increase prenatal care and healthy behaviors during gestation; increase health care access; encourage educational attainment among pregnant women and teen mothers; and provide health care and childcare for infants of young mothers. Educational experts should watch for young mothers who are at risk of dropping out of high school or, less visibly, of forgoing college and other advanced levels of educational attainment. Our findings suggest that reducing social disadvantage is a promising policy route. Such a reduction may simultaneously decrease rates of teen childbearing and of teen mothers’ smoking behaviors, as well as ameliorating the negative socioeconomic consequences of teen childbearing.

At the larger societal level, social and health policies can address unacceptably high rates of unintended pregnancies, poverty, and smoking among teenagers. Compared to older age groups, US children are more likely to live in poverty, with poverty rates of 18.0% for individuals under the age of 18, 11.6% for individuals aged 65 and over, and 9.3% for individuals aged 18 to 64 in 2016 (Semega, Fontenot, and Kollar 2017). More Americans are now insured due in part to the Affordable Care Act, which can provide birth control information to teenagers, prenatal care to pregnant teens, and care to newborns and their mothers. In fact, the percentage of uninsured Americans declined from 16.0% to 9.0% between 2010 and 2016, based on the time of interview with the National Health Interview Survey (Cohen, Zammitti, and Martinez 2017). Thus, changing structural conditions – including increasing health care access, reducing poverty, and improving the quality of schools and neighborhoods – could reduce risky behaviors, including smoking and engaging in risky sex, and thereby improve the quality of life and boost the health of young women and their children.

Smoking is the top preventable cause of death in high-income countries (Mokdad et al. 2004; Rogers et al. 2005), and smoking during pregnancy is a top preventable cause of fetal and infant mortality (Delpisheh, Brabin, and Brabin 2006). Smoking often begins in adolescence, and young adolescent smokers are more likely to become long-term smokers (Woolf and Aron 2013). Smoking can affect the health of teen mothers and their children, and is related to “low birthweight babies, preterm delivery, fetal death, stillbirths, reduced lung function in infants, and sudden infant death syndrome” (Frieden and Blakeman 2005: 1501). Enhanced public health efforts can reduce cigarette advertising and smoking depicted in movies, videos, and on television that appeal to children and adolescents (Frieden and Blakeman 2005). Some cigarette advertising – including the ‘Camel No. 9’ campaign that RJ Reynolds launched in 2007 – is extremely appealing to young girls (Pierce et al. 2010). Because most pregnant women understand the detrimental effects of smoking on their own and their unborn children’s health, they have added motivation to quit and therefore become prime candidates for smoking cessation programs (Delpisheh, Brabin, and Brabin 2006). Compared to other industrialized countries, the United States has relatively low life expectancies and very high rates of teen fertility and such adverse birth outcomes as low birthweight babies, prematurity, and infant mortality (Woolf and Aron 2013). Interventions to reduce teen smoking and fertility could contribute to increased life expectancies and close part of the unacceptably high gap between the United States and its peer countries.

Our results highlight the strengths of the Add Health dataset. One innovative aspect of our analyses is the ability to use multiple waves to obtain smoking information on teen women before they became pregnant and follow them after they bore children. Additional research that builds on our results is merited. For example, future research could expand upon our analyses by examining data from wave 5, which would follow women longer into adulthood and track smoking patterns.

Beyond drawbacks arising from being able to definitively establish time order but not causality, this study has three key limitations. First, although we provided detail on non-Hispanic Whites and Blacks and Hispanics for which we had sufficient sample sizes, future research could focus on other small but important subpopulations, including Asians and Native Americans, as well as on teen fathers. Future research could also examine more detail within the racial/ethnic groups we examined, including country of origin (e.g., comparing Hispanic Mexican Americans, Puerto Ricans, and Cubans) and nativity. Indeed, we found that compared to the foreign-born population, the native-born population was about twice as likely to smoke by wave 4. Second, we could incorporate other factors that may affect the association between pregnancy and smoking, including relief from anxiety and weight control. Finally, while the Add Health data is current, the results may be affected by historical period. Future research could examine other datasets to see if the results respond to temporal and spatial variations.

Our findings underscore the importance of better understanding the relationship between teenage childbirth and subsequent smoking across racial/ethnic groups. Further reducing teen pregnancy could concomitantly reduce low weight and premature births and smoking among young women. Thus, reducing this cluster of risks could improve particularly vulnerable subpopulations, including infants and teen mothers, who may be especially amenable to health interventions. Moreover, investments in teen mothers and infants may pay long-term future dividends in added person-years of health, which will ultimately increase the overall health and longevity of the general population.

## Figures and Tables

**Table 1:** Weighted means for variables used in analyzing daily smoking and teenage motherhood

	All (N = 7,529)	Wave 4 smoker (N = 1,581)	Wave 4 nonsmoker (N = 5,948)	Difference
Smoker at wave 4 (1 = yes)	0.21			
Smoker at wave 1 (1 = yes)	0.11	0.30	0.05	***
Teenage mother (1 = yes)	0.14	0.21	0.11	***
**Demographic characteristics**				
Age at wave 1 (years)	15.80	15.60	15.85	*
**Race**				
Non-Hispanic White *	0.68	0.84	0.64	***
Non-Hispanic Black	0.16	0.08	0.18	***
Hispanic	0.12	0.06	0.14	***
Other	0.04	0.02	0.04	*
Born in the United States (1 = yes)	0.93	0.97	0.92	***
**Selection factors (wave 1)**				
Grade point average				
3.5–4.0 *	0.33	0.21	0.36	***
3.0–3.49	0.27	0.27	0.27	
1.0–2.99	0.40	0.52	0.37	***
Parents’ mean education (years)	13.08	12.68	13.19	***
**Household poverty status (% FPL)**				
>400 *	0.22	0.17	0.23	**
301–400	0.15	0.14	0.15	
201–300	0.21	0.21	0.21	
101–200	0.23	0.27	0.22	*
0–100	0.19	0.2	0.18	
**Family structure**				
Two biological parents *	0.55	0.46	0.58	***
Two parents (other types)	0.17	0.22	0.15	***
Single mom	0.21	0.22	0.21	
Single dad	0.02	0.04	0.02	+
Other family structures	0.05	0.06	0.04	*
**Religious service attendance**				
≥once a week *	0.40	0.31	0.43	***
≥once a month<once a week	0.19	0.18	0.19	
<once a month	0.19	0.21	0.18	*
Never/no religion	0.22	0.30	0.20	***
Logged depression scale	0.45	0.50	0.44	***
Logged delinquency scale	0.19	0.24	0.17	***
History of vaginal intercourse (1 = yes)	0.34	0.48	0.31	***
**Mediators (wave 4)**				
Respondent’s mean education (years)	14.31	13.20	14.60	***
**Household income (% FPL)**				
>400 *	0.37	0.23	0.40	***
301–400	0.12	0.10	0.13	*
201–300	0.21	0.22	0.21	
101–200	0.17	0.23	0.15	***
0–100	0.13	0.21	0.11	***
Home ownership (1 = yes)	0.44	0.38	0.46	***
Logged depression scale	0.46	0.51	0.44	***

*Source:* National Longitudinal Study of Adolescent Health (1995).

*Note:* Reference categories are indicated with * next to variable names. Analyses account for sample design effects (weighting, stratification, and clustering). Depression and delinquency scales are logged after adding 1 to the original scales (0–3).

+ p<.10;

* p<.05;

** p<.01;

*** p<.001.

**Table 2:** Odds ratios from binary logistic regression models predicting wave 4 daily smoking by teenage motherhood, selection factors, mediators, and controls

	N = 7,529				N = 7,036
	Model 1	Model 2	Model 3	Model 4	Model 5
Teen mother (1 = yes)	2.53 ***	1.57 ***	1.47 ***	1.17	1.25
**Demographic characteristics**					
Age at wave 1 (years)	0.96	0.83 ***	0.79 ***	0.83 ***	0.83 ***
**Race/ethnicity (non-Hispanic White)**					
Non-Hispanic Black	0.31 ***	0.20 ***	0.28 ***	0.24 ***	0.28 ***
Hispanic	0.31 ***	0.21 ***	0.26 ***	0.25 ***	0.23 ***
Other	0.55 **	0.50 **	0.60 *	0.58 *	
Born in the United States (1 = yes)	2.33 **	2.25 **	1.96 *	1.77+	1.52
Race* **teen mother**					
Black* teen mother					.50 *
Hispanic* teen mother					1.19
**Selection factors (wave 1)**					
Grade point average (3.5–4.0)					
3.0–3.49		1.59 ***	1.58 ***	1.27+	1.23
1.0–2.99		1.91 ***	1.81 ***	1.23	1.22
Parents’ mean education (years)		0.92 ***	0.93 **	0.98	0.98
**Household income (% FPL) (>400)**					
301–400		1.24	1.25	1.20	1.19
201–300		1.19	1.19	1.06	1.06
101–200		1.38+	1.39+	1.15	1.13
0–100		1.40+	1.37	1.01	0.99
**Family structure (2 biological parents)**					
2 parents (other types)		1.48 ***	1.46 **	1.33 *	1.32 *
Single mom		1.22+	1.17	1.15	1.16
Single dad		1.75+	1.59	1.52	1.54
Other family structures		1.75 **	1.60 *	1.32	1.38+
**Religious service attendance (≥once a week)**					
≥ once a month < once a week		1.13	1.07	1.07	1.05
< once a month		1.12	0.96	0.92	0.89
Never/no religion		1.37 **	1.22+	1.12	1.11
Logged depression scale		1.37+	1.28	0.93	0.92
Logged delinquency scale		2.22 ***	1.54+	1.58 *	1.62 *
History of vaginal intercourse (1 = yes)		2.11 ***	1.65 ***	1.64 ***	1.63 ***
Smoker at wave 1 (1 = yes)			4.70 ***	4.44 ***	4.42 ***
**Mediators (wave 4)**					
Respondent’s mean education (years)				0.82 ***	0.82 ***
**Household poverty status (%FPL) (>400)**					
301–400				1.15	1.14
201–300				1.25+	1.28+
101–200				1.64 **	1.58 **
0–100				1.78 ***	1.77 ***
Home ownership (1 = yes)				0.72 **	0.71 **
Logged depression scale				1.47 **	1.47 **
Constant	0.28 *	2.09	4.55 *	25.24 ***	30.28 ***

*Source:* National Longitudinal Study of Adolescent Health (1995).

*Note:* Models 1–4 include all racial/ethnic groups (N = 7529); model 5 includes White, Black, and Hispanic (N = 7036). Analyses account for sample design effects (weighting, stratification, and clustering).

+ p<.10;

* p<.05;

** p<.01;

*** p<.001.

**Table 3:** Odds ratios from binary logistic regression models predicting daily smoking at wave 4 by teenage motherhood, disaggregated by race/ethnicity

	Model 1	Model 2	Model 3	Model 4
Teen mother among Whites (n = 4,163)	2.87 ***	1.68 ***	1.56 ***	1.23
Teen mother among Blacks (n = 1,681)	0.94	0.70	0.70	0.62
Teen mother among Hispanics (n = 1,191)	2.65 ***	1.94+	1.82+	1.23

*Source:* National Longitudinal Study of Adolescent Health (1995).

*Note:* DV: daily smoking at wave 4, IV: teenage motherhood, models restricted to each racial/ethnic group. Model 1: controls for demographic characteristics; model 2: controls for demographic characteristics and selection factors; model 3: controls for demographic characteristics, selection factors, and smoking at wave 1; model 4: controls for demographic characteristics, selection factors, smoking at wave 1, and mediators. Analyses account for sample design effects (weighting, stratification, and clustering).

+ p<.10;

* p<.05;

** p<.01;

*** p<.001.

## Footnotes

4 Among the 345 respondents who were excluded from analysis because of pregnancy or childbirth prior to wave 1, 21.1% and 37.6% were daily smokers at waves 1 and 4, respectively.

5 Of the variables used for the 20 imputed datasets, wave 1 household income (24.9%), nativity (19.8%), and GPA (16.2%) had the highest percent of missing information among the eligible cases. Sensitivity analyses that compare imputed to nonimputed results did not show substantive differences in conclusions drawn, with the exception of racial/ethnic heterogeneity in selection and mediation processes. Compared to imputed analyses, nonimputed analyses showed similar results among Black women, a greater association between teen childbearing and subsequent smoking that was mediated by achieved income among Hispanic women, and the disappearance of the significant association between teen childbearing and subsequent smoking once selection variables were included in the model among White women.

6 This variable was coded as: 8th grade or less = 8 years, some high school = 10, high school graduate = 12, some vocational/technical training (after high school) = 12.5, completed vocational/technical training (after high school) = 13, some college = 14, completed college (bachelor’s degree) = 16, some graduate school = 17, completed a master’s degree = 18, some graduate training beyond a master’s degree = 19, completed a doctoral degree = 20, some post baccalaureate professional education (e.g., law school, med school, nurse) = 19, and completed post baccalaureate professional education (e.g., law school, med school, nurse) = 20 years.
